# Properties of the Geometric Phase in Electromechanical Oscillations of Carbon-Nanotube-Based Nanowire Resonators

**DOI:** 10.1186/s11671-019-2855-8

**Published:** 2019-02-05

**Authors:** Jeong Ryeol Choi, Sanghyun Ju

**Affiliations:** Department of Physics, Kyonggi University, Gwanggyosan-ro, Yeongtong-gu, Suwon, Gyeonggi-do, 16227 Republic of Korea

**Keywords:** Geometric phase, Nanowire resonator, Electromechanical oscillation, Squeezed state, Invariant operator, Phase effect

## Abstract

The geometric phase is an extra phase evolution in the wave function of vibrations that is potentially applicable in a broad range of science and technology. The characteristics of the geometric phase in the squeezed state for a carbon-nanotube-based nanowire resonator have been investigated by means of the invariant operator method. The introduction of a linear invariant operator, which is useful for treating a complicated time-dependent Hamiltonian system, enabled us to derive the analytical formula of the geometric phase. By making use of this, we have analyzed the time behavior of the geometric phase based on relevant illustrations. The influence of squeezing parameters on the evolution of the geometric phase has been investigated. The geometric phase, in large, oscillates, and the envelope of such oscillation increases over time. The rate of the increase of the geometric phase is large when the parameters, such as the classical amplitude of the oscillation, the damping factor, and the amplitude of the driving force, are large. We have confirmed a very sharp increase of the geometric phase over time in the case that the angular frequency of the system reaches near the resonance angular frequency. Our development regarding the characteristics of the geometric phase is crucial for understanding the topological features in nanowire oscillations.

## Introduction

Mechanical vibrations of the smallest resonators, such as carbon-nanotube-based (CNT-based) nanowires [1–3], semiconducting nanowires [4], graphenes [5], and levitated particles [6], have been a main research subject in the nanoscience community for over a decade. Active researches regarding electromechanical oscillations of nanowire resonators driven by an external periodic force have been performed in both theoretical and experimental spheres. In particular, CNT-based nanowire resonators have attracted considerable interest as nanoscale mechanical devices due to their extraordinary sensitivities with high-quality factors to a small perturbation from surroundings. Suspended CNT-based nanowire resonators are promising candidates for apparatuses measuring a wide range of physical quantities, such as EM waves [2], small forces [7], masses [8], temperatures [9], and noises [10].

Analyses of the quantal phase evolution in nanowire oscillations are required for elucidating underlying features of the system theoretically. Regarding quantum vibrational states of the CNT-based nanowire resonators [11], the geometric phase [12] as well as the usual dynamical phase emerges as a supplementary evolution of the phase. The geometric phase [12] is an anholonomic of a quantum state which can be applicable in diverse fields of physics. Analyses of the geometric phase can be potentially adopted in characterizing nano properties of nanowires, such as the resonance profiles [13, 14], strong quantum vibrations [15, 16], strain relaxation mechanisms [17, 18], the emergence of Dirac magnetoplasmons [19], and the topology of Aharonov-Bohm oscillations [20].

The study of the geometric phase associated with nonadiabatic dynamics may provide an insight for nanomechanical systems, which is necessary for the advancement of accurate simulation techniques [21]. The preparation, manipulation, and detection of quantum states are important factors in quantum technologies. The aim of the present research is to shed light on time behaviors of the geometric phase that takes place in quantum states of nanowire oscillations. To understand the mechanism of CNT-based nanowire vibrations, we will investigate the time evolution of the geometric phase in the squeezed state which is a classical-like quantum state like the coherent state. The merit of the squeezed state is that the uncertainty of a quadrature in that state can be reduced substantially at the expense of rising the uncertainty of the other quadrature, while such uncertainty modulation is impossible in the coherent state. In particular, we will analyze the effects of resonance on the geometric phase. Because the resonant energy is significantly different from the energy of the non-resonant state [22, 23], the topological behavior of the wave function is nontrivial and may be considerably deviated from the one in normal situations. The influence of the change of physical parameters and the squeezing parameters on the evolution of the geometric phase will also be rigorously analyzed. The geometric phases are ubiquitous in dynamical systems [24] and can be applied to various modern technologies, such as quantum computation [25], intensity interferometries [26], photonic multitasking [27], quantum-sensing protocols [28], and wave-stability measurements [29].

The Hamiltonian of the system involves time functions associated with the damping of the system and the external driving force. Hence, the system is a kind of time-dependent Hamiltonian systems (TDHSs) of which quantum mechanical problems are extensively studied up to recently. The time function in the Hamiltonian of a TDHS cannot be separated out from the function of canonical variables in most cases, leading the conventional separation of variables method for solving the Schrödinger equation being unavailable. An alternative powerful method developed for overcoming this difficulty is the invariant operator method which has been introduced by Lewis and Riesenfeld [30, 31]. This method is a very useful mathematical tool when we derive quantum solutions of a TDHS. Many quantum mechanical problems described by TDHSs are investigated based on this method. For instance, they include chaotic particle-scattering [32], light propagation in time-varying media [33], control of trapped driven electrons [34], and nonclassicality of quantum nanoelectronic circuits [35]. There is a variety of other methods for quantum mechanical treatments of TDHSs, which include unitary transformation method [36], Lie algebraic method [37], and Hamiltonian estimation method [38].

Regarding that the system is a TDHS, we use the invariant operator method in order to obtain quantum solutions of the system. A linear invariant operator which is represented in terms of the annihilation operator will be introduced. While the annihilation and the creation operators are represented in terms of time due to the time-dependence of the system, both the coherent and the squeezed states can be obtained using these ladder operators. The geometric phase of the system will be analytically evaluated by utilizing the wave function in the squeezed state. The time evolution of the geometric phase will be analyzed in detail on the basis of its illustrations depicted with diverse choices of parameters.

## Methods

To investigate the geometric phase, we first need to setup the classical equation of motion of the nanowire tip. Because the geometric phase appears in the quantum wave evolution of a TDHS, it is necessary to derive wave functions in a specific quantum state that we manage. We will consider the squeezed state as mentioned in the introductory part. The wave functions in the diverse quantum states of a TDHS, including the squeezed state, can be obtained from the invariant operator method.

The equation of motion for the time-dependent amplitude *x* for a bending mode of a suspended carbon nanotube with an effective mass *m* is given by [1]

1 $$ \ddot{x}+\left(\frac{\omega_{0}}{Q} +\eta x^{2}\right) \dot{x}+\left(\omega_{0}^{2}+\beta x^{2}\right) x = f_{\mathrm{d}}\cos (\omega t),   $$

where *ω*_0_ is the resonant angular frequency, *Q* the quality factor, *f*_d_ the electrostatic driving force divided by *m*, *η* the nonlinear damping coefficient, and *β* the Duffing parameter. Let us assume for convenience that the displacement of the tip is sufficiently small relative to the CNT-wire length. Then, we can neglect the nonlinear terms in Eq. (1), leading to [2]

2 $$ \ddot{x}+\frac{\omega_{0}}{Q} \dot{x}+\omega_{0}^{2} x = f_{\mathrm{d}}\cos (\omega t).  $$

The Hamiltonian of the system which yields Eq. (2) is given by

3 $$ \hat{H}= e^{-\gamma t} \frac{\hat{p}^{2}}{2m} +\frac{1}{2}me^{\gamma t} \left[\omega_{0}^{2} \hat{x}^{2} - 2f_{\mathrm{d}}\cos (\omega t)\hat{x}\right],   $$

where *γ*=*ω*_0_/*Q*. The classical solution of Eq. (2) is composed of a complementary function *X*_*c*_(*t*) and a particular solution *X*_*p*_(*t*), which are given by

4 $$\begin{array}{@{}rcl@{}} & &X_{c}(t)=X_{c,0}e^{-\gamma t/2}\cos(\Omega t+\varphi),  \end{array} $$

5 $$\begin{array}{@{}rcl@{}} & &X_{p}(t) =X_{p,0}\cos (\omega t - \delta),  \end{array} $$

where *X*_*c*,0_ is a constant, $\Omega = \sqrt {\omega _{0}^{2} - \gamma ^{2}/4}$, *φ* is an arbitrary phase, and

6 $$\begin{array}{@{}rcl@{}} X_{p,0}&=&\frac{f_{\mathrm{d}}}{\sqrt{\left(\omega_{0}^{2} -\omega^{2}\right)^{2} + \gamma^{2} \omega^{2}}},  \end{array} $$

7 $$\begin{array}{@{}rcl@{}} \delta &=& \tan^{-1} \frac{\gamma \omega}{ \omega_{0}^{2} -\omega^{2}}.  \end{array} $$

The classical solution in the momentum space is given in a similar way, where the complementary function is $P_{c} (t) = m e^{\gamma t} \dot {X}_{c}(t)$ and the particular solution is $P_{p} (t) = m e^{\gamma t} \dot {X}_{p}(t)$. To investigate the geometric phase of the system, we first need to derive quantum solutions. Notice that the Hamiltonian of the system given in Eq. (3) is explicitly dependent on time. In order to derive quantum solutions of the system, we use the invariant operator method [30, 31], which is a useful method when we treat such a time-varying system. An invariant operator $\hat {I}$ of the system can be derived from the Liouville-von Neumann equation, which is given by ${d \hat {I}}/{d t} = {\partial \hat {I}}/{\partial t} + \left [\hat {I},\hat {H}\right ]/\left (i\hbar \right) = 0$. Hence, from a rigorous evaluation after inserting Eq. (3) into this equation, we have a linear invariant operator [34] of the form

8 $$ \hat{I} = \hat{A} e^{i\Omega t},  $$

where $\hat {A}$ is the annihilation operator that is given by

9 $$ \begin{aligned} \hat{A} =& \left(2\hbar m\Omega\right)^{-1/2} \left[ m \left(\Omega+ i\frac{\gamma}{2} \right) e^{\gamma t/2}\left[\hat{x}-X_{p}(t)\right]\right.\\ & \left.+ie^{-\gamma t/2} \left[\hat{p}-P_{p}(t)\right]\! {\vphantom{\left(\Omega+ i\frac{\gamma}{2} \right)}}\right]. \end{aligned}  $$

The hermitian adjoint of Eq. (9), $\hat {A}^{\dagger }$, is the creation operator.

We can express the eigenvalue equation of $\hat {A}$ as

10 $$ \hat{A} |A \rangle = A |A \rangle.  $$

By evaluating the above equation, we have the expression of the eigenvalue such that

11 $$ A(t) = A(0) e^{-i\Omega t},  $$

where *A*(0)=*A*_0_*e*^−*iφ*^ with

12 $$ A_{0} = \left[m\Omega/(2\hbar)\right]^{1/2}X_{c,0}.  $$

While the coherent state |*A*〉 is the eigenstate of $\hat {A}$, the squeezed state is the eigenstate of an operator $\hat {B}$ that is given by

13 $$ \hat{B} = \mu \hat{A} + \nu \hat{A}^{\dagger},  $$

where *μ* and *ν* are complex variables that yield the equation

14 $$ |\mu|^{2} - |\nu|^{2} =1.  $$

If we write the eigenvalue equation of $\hat {B}$ in the form

15 $$ \hat{B} |B \rangle = B |B \rangle,  $$

|*B*〉 is the squeezed state. By solving this equation in the configuration space, we have

16 $$ {\begin{aligned} \langle {x}|B\rangle =&^{4}\!\!\!\sqrt{\frac{m \Omega e^{\gamma t}}{\hbar\pi(\mu-\nu)(\mu^{*}-\nu^{*})}} \exp \left\{- \frac{1}{\hbar (\mu-\nu)} \left[\frac{1}{2} m e^{\gamma t}\left({\vphantom{\frac{1}{2}}}(\mu+\nu)\Omega \right.\right.\right.\\ & \left. +\frac{i\gamma}{2}(\mu-\nu)\right)\left[x-X_{p}(t)\right]^{2} -[iP_{p}(t)(\mu-\nu)+ \left(2\hbar m \Omega e^{\gamma t}\right)^{1/2} \\ & \left. \left.\times(\mu A+\nu A^{*}) ]\left[x-X_{p}(t)\right] {\vphantom{\frac{1}{2} m e^{\gamma t}}}\right]-\frac{|A|^{2}+A^{2}}{2(\mu-\nu)(\mu^{*}-\nu^{*})} \right\}. \end{aligned}}   $$

Thus, the wave function in the squeezed state has been derived as given in Eq. (16). Quantum features of the system can be clarified on the basis of such analytical description of the wave function. For *μ*=1 and *ν*=0, Eq. (16) reduces to the wave function in the coherent state, which is the eigenstate of Eq. (10) in the configuration space. The wave function, Eq. (16), will be used in the next section in order to derive the geometric phase in the squeezed state.

## Results and Discussion

It is well known that the phase in the quantum wave evolution involves the geometric phase as well as the dynamical phase. The geometric phase was first discovered by Berry in 1984 [12] for a system evolving cyclically with an adiabatic change. According to the adiabatic theorem in quantum mechanics, an instantaneous eigenstate of a quantum state in a cyclic evolution in the parameter space will remain on the same state later, while there is an additional accumulation of the quantum phase which is the Berry phase. A generalization of the Berry phase in a way that it includes nonadiabatic, noncyclic, and/or non-unitary evolution of the quantum system is the geometric phase.

The geometric phase in the squeezed state is given by

17 $$ \gamma_{G}(t) = \int_{0}^{t} \langle B(t') |i\frac{\partial}{\partial t'}| B(t') \rangle dt' +\gamma_{G}(0).  $$

The differentiation of the wave function with respect to time in configuration space becomes

18 $$ \frac{\partial \langle {x}|B\rangle}{\partial t} \,=\, \left\{ f_{1}(t) \!\left[x-X_{p}(t)\right]^{2}\,+\,f_{2}(t) \left[x\,-\,X_{p}(t)\right]\,+\,f_{3}(t) \right\}\! \!\langle {x}|B\rangle,  $$

where

19 $$ f_{1}(t) = - \frac{m\gamma e^{\gamma t}}{2\hbar (\mu-\nu)} \left((\mu+\nu)\Omega + \frac{i\gamma}{2}(\mu-\nu) \right),  $$

20 $$ {\begin{aligned} f_{2}(t) &= \frac{1}{\hbar (\mu-\nu)}\left[ \left((\mu+\nu)\Omega + \frac{i\gamma}{2}(\mu-\nu) \right) P_{p}(t) -i m e^{\gamma t} \right.\\ & \quad\times\left[\omega_{0}^{2} X_{p}(t) - f_{\mathrm{d}} \cos(\omega t)\right](\mu-\nu) +\left(2\hbar m \Omega e^{\gamma t}\right)^{1/2} \\ & \quad \left.\times\left(\frac{\gamma}{2}\left(\mu A + \nu A^{*}\right)-i\Omega \left(\mu A - \nu A^{*}\right) \right) \right],  \\ \end{aligned}}  $$

21 $$ {\begin{aligned} f_{3}(t) &\!= \frac{\gamma}{4}-\frac{1}{\hbar m e^{\gamma t}(\mu-\nu)} \left[iP_{p}(t)(\mu-\nu) + \left(2\hbar m\Omega e^{\gamma t}\right)^{1/2} \right.\\ & \quad\left.\times\left(\mu A+\nu A^{*}\right){\vphantom{\left(2\hbar m\Omega e^{\gamma t}\right)^{1/2}}}\right] P_{p}(t)+ \frac{i\Omega A^{2}}{(\mu-\nu)\left(\mu^{*}-\nu^{*}\right)}.  \end{aligned}}  $$

Further evaluation after inserting Eq. (18) into Eq. (17) gives

22 $$ {\begin{aligned} \gamma_{G}(t) =& \int_{0}^{t} dt' \left[ A_{0}^{2}\left(\frac{\gamma^{2}}{4\Omega}+\Omega + g_{1} \sin\left[2\left(\Omega t'+\varphi\right)\right] +g_{2} \cos\left[2\left(\Omega t'+\varphi\right)\right] \right) \right.\\ &\left.-A_{0}\left[ g_{3}(t') \sin\left(\Omega t'+\varphi\right) +g_{4}(t') \cos\left(\Omega t'+\varphi\right) \right]+ g_{5}(t') {\vphantom{\frac{\gamma^{2}}{4\Omega}}}\right] +\gamma_{G}(0), \end{aligned}}  $$

where

23 $$\begin{array}{*{20}l} g_{1}~ &= \frac{\gamma}{2} + \frac{i\Omega \left(\mu\nu^{*}-\mu^{*}\nu\right)}{(\mu-\nu)\left(\mu^{*}-\nu^{*}\right)},  \end{array} $$

24 $$\begin{array}{*{20}l} g_{2}~ &= \frac{\gamma^{2}}{4\Omega}+\Omega\frac{2|\nu|^{2}- \left(\mu\nu^{*}+\mu^{*}\nu\right)}{(\mu-\nu) \left(\mu^{*}-\nu^{*}\right)},  \end{array} $$

25 $$\begin{array}{*{20}l} g_{3}(t) &= \left(\frac{2\Omega}{m\hbar e^{\gamma t}} \right)^{1/2}P_{p}(t),  \end{array} $$

26 $$ {\begin{aligned} g_{4}(t) = \frac{1}{\sqrt{2\hbar\Omega}}\left(\frac{\gamma }{\sqrt{m e^{\gamma t}}}P_{p}(t) - 2\sqrt{m e^{\gamma t}}\left[\omega_{0}^{2} X_{p}(t) - f_{\mathrm{d}} \cos(\omega t)\right]\right), \end{aligned}}  $$

27 $$ {\begin{aligned} g_{5}(t) &= \frac{P_{p}^{2}(t)}{\hbar m e^{\gamma t}}+\frac{\gamma^{2}}{8\Omega}\left[2|\nu|^{2}-\left(\mu\nu+\mu^{*}\nu^{*}\right) +1\right] \\ & \quad +\frac{i\gamma}{4(\mu-\nu)\left(\mu^{*}-\nu^{*}\right)} \left[|\mu|^{2}\left(\nu^{2}-\nu^{*2}\right)-|\nu|^{2}\left(\mu^{2}-\mu^{*2}\right)\right.\\ & \quad\left.+ (2|\nu|^{2}+1)\left(\mu\nu^{*}-\mu^{*}\nu\right) +(\mu-\mu^{*})(\nu-\nu^{*})\right].  \end{aligned}}  $$

The last term in *g*_5_ that contains (*μ*−*μ*^∗^)(*ν*−*ν*^∗^) is inadequate as a phase because this is a purely imaginary number. Hence, we now remove this term by choosing at least one of *μ* and *ν* as a real value. This remedy can always be done without loss of generality, because only the relative phase between *μ* and *ν* has physical meaning rather than their absolute phases.

From the execution of the integration in Eq. (22), we have

28 $$ {\begin{aligned} \gamma_{G}(t) &= A_{0}^{2}\left[\left(\frac{\gamma^{2}}{4\Omega}+\Omega\right)t + \frac{g_{1}}{\Omega}\sin(\Omega t+2\varphi) \sin(\Omega t) +\frac{g_{2}}{\Omega} \cos(\Omega t+2\varphi) \right.\\ & \quad\left.\times\sin(\Omega t) {\vphantom{\frac{\gamma^{2}}{4\Omega}}}\right]\!-A_{0}\left[ \left(\frac{2m\Omega}{\hbar} \right)^{1/2}\omega X_{p,0} \bar{g}_{3}(t) +\sqrt{\frac{2m}{\hbar\Omega}}\frac{1}{4\omega^{2}+\gamma^{2}}\bar{g}_{4}(t) \right]\\ &\quad+ \bar{g}_{5}(t) +\gamma_{G}(0),  \end{aligned}}  $$

where $\bar {g}_{i}(t)~(i=3,4,5)$ are given by

29 $$ \bar{g}_{i}(t) = G_{i}(t) -G_{i}(0),  $$

with

30 $$ {\begin{aligned} G_{3}(\tau) &= e^{\gamma \tau/2}\left(\frac{1}{4(\Omega+\omega)^{2}+\gamma^{2}} \left\{2(\Omega+\omega)\sin[(\Omega+\omega)\tau+\varphi-\delta] \right.\right.\\ & \quad\left.+\gamma \cos[(\Omega+\omega)\tau+\varphi-\delta] \right\}- \frac{1}{4(\Omega-\omega)^{2}+\gamma^{2}} \{ 2(\Omega-\omega) \\ & \quad\left.\left.\times\sin[(\Omega-\omega)\tau\,+\,\varphi\,+\,\delta]\!+\gamma \cos[(\Omega-\omega)\tau\,+\,\varphi\,+\,\delta]\right\} {\vphantom{\frac{1}{4(\Omega+\omega)^{2}+\gamma^{2}}}}\right),\\ \end{aligned}}  $$

31 $$ {\begin{aligned} G_{4}(\tau) &= e^{\gamma \tau/2} \left\{X_{p,0} \left\{ \gamma\omega[ 2\omega\cos(\omega \tau-\delta)-\gamma \sin(\omega \tau-\delta)] -2\omega_{0}^{2} \right.\right.\\ &\quad\left.\times[2\omega\sin(\omega \tau-\delta)+\gamma \cos(\omega \tau-\delta)] {\vphantom{X_{p,0}}}\right\}+2f_{\mathrm{d}} [ 2\omega\sin(\omega \tau) \\ & \left.\left.\quad+\gamma \cos(\omega \tau)\right]{\vphantom{X_{p,0}}}\right\}, \\ \end{aligned}}  $$

32 $$ {\begin{aligned} G_{5}(\tau) &= \frac{m\omega^{2}}{2\hbar}X_{p,0}^{2} \frac{e^{\gamma \tau}}{\gamma \left(4\omega^{2}+\gamma^{2}\right)} \left\{ \gamma^{2}+4\omega^{2} -\gamma^{2} \cos[2(\omega\tau -\delta)]\right.\\ & \quad\left.-2\gamma\omega \sin[2(\omega \tau -\delta)] {\vphantom{\gamma^{2}+4\omega^{2} -\gamma^{2}}}\right\} +\frac{\gamma^{2} \tau}{8\Omega}\left[2|\nu|^{2}-\left(\mu\nu+\mu^{*}\nu^{*}\right)+1\right] \\ & \quad+\frac{i\gamma \tau}{4(\mu-\nu)\left(\mu^{*}-\nu^{*}\right)} \left[|\mu|^{2}\left(\nu^{2}-\nu^{*2}\right)-|\nu|^{2}\left(\mu^{2}-\mu^{*2}\right)\right.\\ & \quad\left.+\left(2|\nu|^{2}+1\right)\left(\mu\nu^{*}-\mu^{*}\nu\right)\right].  \end{aligned}}  $$

Thus, we have evaluated the full geometric phase in the squeezed state, which is given by Eq. (28) with Eqs. (23), (24), and (29)–(32).

**Fig. 1:**
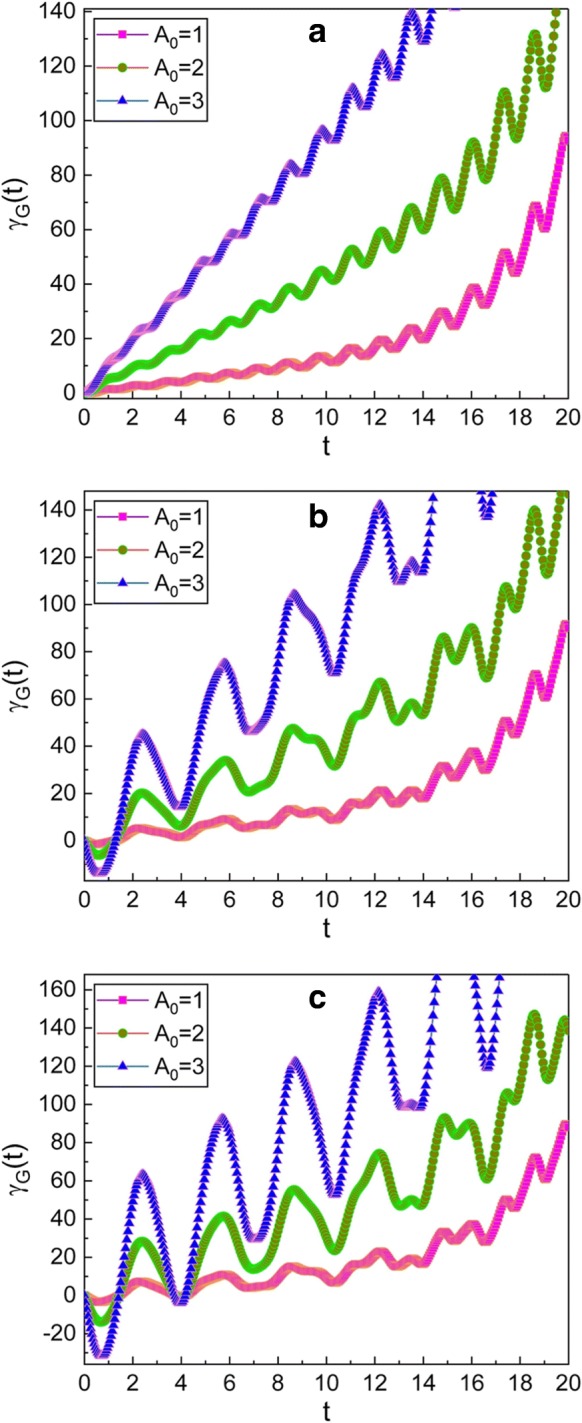
Time evolution of the geometric phase for several different values of *A*_0_. The values of (*μ*, *ν*) used in the graphics are (1, 0) for **a**, ($\sqrt {2}$, 1) for **b**, and ($\sqrt {3}$, $\sqrt {2}$) for **c**. We have used *m*=1, *ω*_0_=1, *ω*=5, *γ*=0.35, *f*_d_=1, $\hbar =1$, *φ*=0, and *γ*_*G*_(0)=0. The phase and all parameters are taken to be dimensionless for convenience, and this convention will also be applied to the subsequent figures. Because *A*_0_ is given in terms of the classical amplitude *X*_*c*,0_ of the complementary function [see Eq. (12)], we can confirm from the graphics that the geometric phase is large when the oscillation amplitude is high. We also see that the fluctuation of *γ*_*G*_(*t*) becomes large as the values *μ* and *ν* increase under the condition given in Eq. (14)

**Fig. 2:**
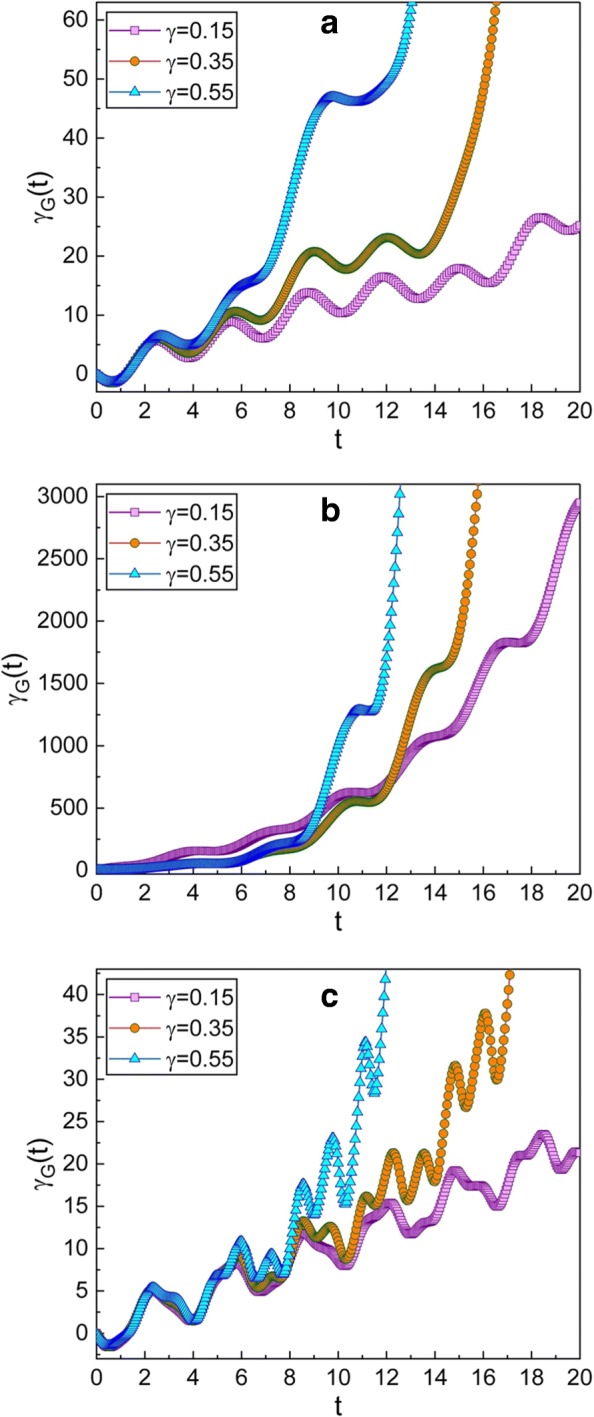
Time evolution of the geometric phase for several different values of *γ*. The value of *ω* used in the graphics is 0.3 for **a**, 0.99 for **b**, and 5 for **c**. The squeezing parameters chosen here are $\mu =\sqrt {2}$ and *ν*=1; this choice gives *q*-squeezed state at initial time. Other quantities that we have used are *m*=1, *ω*_0_=1, *A*_0_=1, *f*_d_=1, $\hbar =1$, *φ*=0, and *γ*_*G*_(0)=0. We confirm that the geometric phase is large when the damping factor *γ* is large in most cases, but not all. The frequency of the case **b** is near to the resonant frequency, whereas those of **a** and **c** are far from the resonant one. The geometric phase for the resonant case (**b**) increases very rapidly over time

**Fig. 3:**
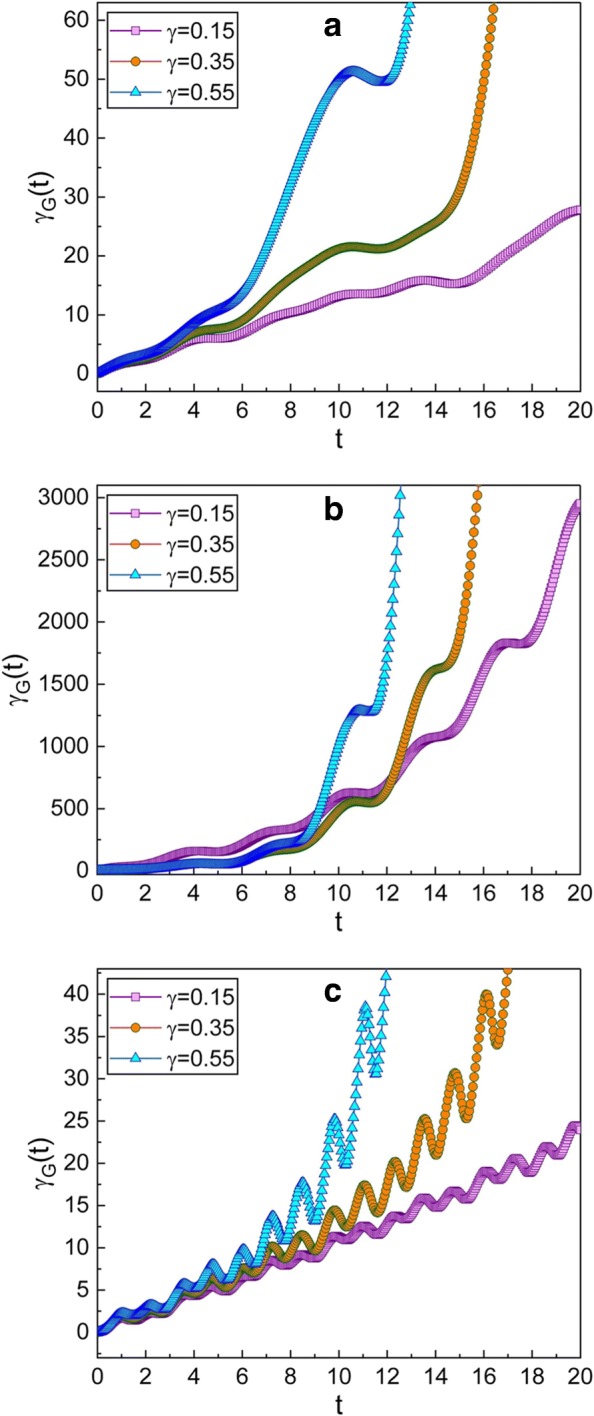
**a**–**c** This graphic is the same as Fig. 2, but for the case that the chosen squeezing parameters are $\mu =\sqrt {2}$ and *ν*=−1 which give a *p*-squeezed state at initial time. From the fact that the overall graphics in this case are not so much different from the corresponding ones of Fig. 2, we can confirm that the evolution of *γ*_*G*_(*t*) is nearly irrelevant to the types of squeezing so long as the absolute values of *μ* and *ν* do not change

**Fig. 4:**
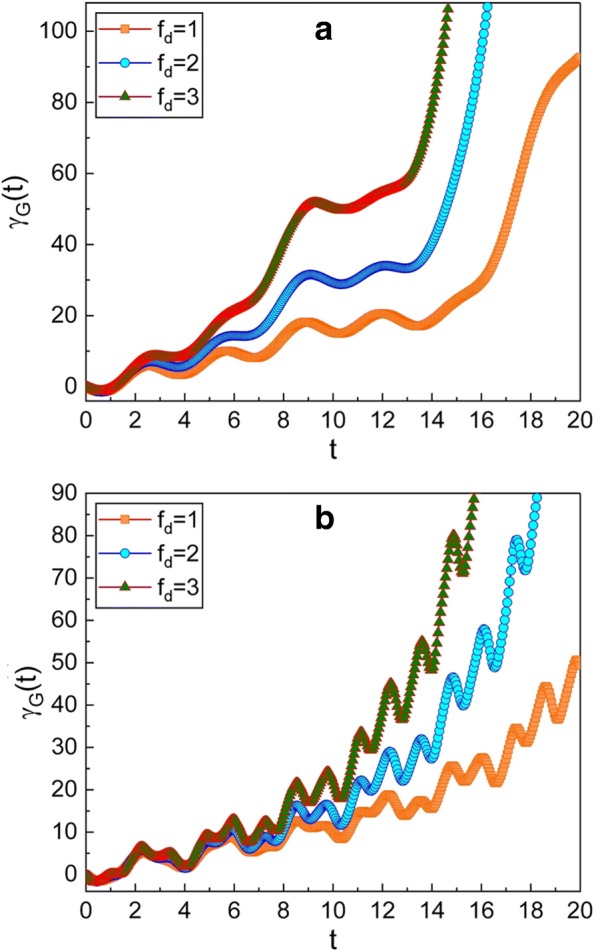
Time evolution of the geometric phase for several different values of *f*_d_. The value of *ω* used in the graphics is 0.3 for **a** and 5 for **b**. We have used $\mu =\sqrt {2}$, *ν*=1, *m*=1, *ω*_0_=1, *γ*=0.3, *A*_0_=1, $\hbar =1$, *φ*=0, and *γ*_*G*_(0)=0. As the amplitude (*f*_d_) of the driving force increases, the geometric phase becomes large

The time evolution of the geometric phase has been illustrated in Figs. 1, 2, 3, and 4. From Fig. 1, we see that the geometric phase oscillates and the envelope of such oscillation increases over time. The increase of the envelope is greater when *A*_0_ is large. The pattern of the oscillation becomes gradually irregular as the values of *μ* and *ν* increase. Moreover, the amplitude of the oscillation becomes large as time goes by.

The squeezing effects in the squeezed state depending on the squeeze parameter *c* where *c*=*μ*/*ν* has been investigated in ref. [39]. According to the analysis given in ref. [39] (see Fig. 1(a) in ref. [39]), the squeezed state illustrated in Fig. 2, which corresponds to $c=\sqrt {2}$, is the *q*-squeezed state at initial time, while that in Fig. 3, which corresponds to $c=-\sqrt {2}$, is the *p*-squeezed state in the same situation. By comparing Figs. 2 and 3 to each other, we can conclude that the geometric phase in the *q*-squeezed state is nearly the same as that in the *p*-squeezed state.

The effects of *γ* on the evolution of the geometric phase can be confirmed from Figs. 2 and 3. The geometric phase increases more rapidly when *γ* is large. By comparing Figs. 2a and 3a with Figs. 2c and 3c, we can confirm that the geometric phase varies somewhat rapidly when *ω* is greater than the resonance angular frequency.

The time behavior of the geometric phase at or near the resonant state of the system may be of great interest [22, 23]. Figures 2b and 3b show that the geometric phase increases very rapidly when *ω* is near the resonance angular frequency. This means that the wave function in this situation varies significantly over time, because the magnitude of the geometric phase is related to the time variation of the wave function. As a matter of fact, the amplitude of the wire oscillation is remarkably augmented at the resonance state. By the way, resonance angular frequencies of suspended CNT-based nanowire resonators are not only high but also widely tunable with very high-quality factors [3]. For this reason, the vibrational modes of the system will be kept for a long time until they thoroughly damped out [11].

Figure 4 shows that the geometric phase is also affected by the amplitude of the driving force *f*_d_. As *f*_d_ increases, the increment of the geometric phase in time is rapid.

## Conclusion

We have investigated the geometric phase in the squeezed state for the system on the basis of quantum dynamics with the Schrödinger equation. Regarding time-dependence of the Hamiltonian that describes the system, the invariant operator method has been introduced, which is a potential tool for deriving quantum solutions in the case where the Hamiltonian is described in terms of time. By means of this method, the analytical formula of the geometric phase for the CNT-based nanowire oscillation has been obtained.

A detailed analysis of the phase effects, which is necessary for a theoretical understanding of the mechanical vibrations, has been carried out. Our development of the geometric phase is a fully quantum-based one with rigorous mathematical evaluations. The geometric phase is sensitive to the change of mechanical parameters and exhibits an oscillation in a large number of cases. The influence of the squeezing parameters on the evolution of the geometric phase has also been analyzed. We have confirmed a strong increase of geometric phase accumulation over time near the resonant angular frequency.

Our results illustrate the time behavior of the geometric phase that appears in the vibration of a CNT-based nanowire. The analysis of the geometric phase given in this work is important for understanding not only topological features of the system but dynamical vibrations of other nanowire-based mechanical oscillators as well. In particular, we have developed phase properties of the resonant state, of which clarification is necessary in the application of the system in quantum information technologies and other quantum-based industries [40]. The similar method and framework used in this research can also be extended to other nano systems, such as superconducting Fabry-Perot resonators [41], nano cantilevers [42], and qubit-resonator-atom hybrid systems [43].

## Abbreviations

CNT: Carbon nanotube
EM waves: Electromagnetic waves
TDHS: Time-dependent Hamiltonian system
